# Cysteinyl leukotriene signaling through perinuclear CysLT_1_ receptors on vascular smooth muscle cells transduces nuclear calcium signaling and alterations of gene expression

**DOI:** 10.1007/s00109-012-0904-1

**Published:** 2012-04-20

**Authors:** Alison Eaton, Edit Nagy, Mathilde Pacault, Jérémy Fauconnier, Magnus Bäck

**Affiliations:** 1 Department of Medicine, Karolinska Institutet, Stockholm, Sweden; 2 Department of Physiology & Pharmacology, Karolinska Institutet, Stockholm, Sweden; 3 INSERM U1046, Université Montpellier 1, Université Montpellier 2, Montpellier, France; 4 Center for Molecular Medicine L8:03, Karolinska University Hospital, 171 76 Stockholm, Sweden

**Keywords:** Atherosclerosis, Eicosanoids, Inflammation, Lipoxygenase, PAI-2

## Abstract

Leukotrienes are pro-inflammatory mediators that are locally produced in coronary atherosclerotic plaques. The response induced by cysteinyl leukotrienes (CysLT) in human coronary arteries may be altered under pathological conditions, such as atherosclerosis. The aim of the present study was to elucidate cysteinyl leukotriene signaling in vascular smooth muscle cells (SMCs) and the effects of inflammation on this process. Immunohistochemical analysis of human carotid endarterectomy samples revealed that the CysLT_1_ leukotriene receptor was expressed in areas that also stained positive for α-smooth muscle actin. In human coronary artery smooth muscle cells, lipopolysaccharide significantly upregulated the CysLT_1_ receptor and significantly enhanced the changes in intracellular calcium induced by leukotriene C_4_ (LTC_4_). In these cells, the CysLT_1_ receptor exhibited a perinuclear expression, and LTC_4_ stimulation predominantly enhanced nuclear calcium increase, which was significantly inhibited by the CysLT_1_ receptor antagonist MK-571. Microarray analysis revealed, among a number of significantly upregulated genes after 24 h stimulation of human coronary artery smooth muscle cells with LTC_4_, a 5-fold increase in mRNA levels for plasminogen activator inhibitor (PAI)-2. The LTC_4_-induced increase in PAI-2 expression was confirmed by real-time quantitative PCR and ELISA and was inhibited by the CysLT_1_ receptor antagonist MK-571 and by calcium chelators. In summary, pro-inflammatory stimulation of vascular SMCs upregulated a perinuclear CysLT_1_ receptor expression coupled to nuclear calcium signaling and changes in gene expression, such as upregulation of PAI-2. Taken together, these findings suggest a role of nuclear CysLT_1_ receptor signaling in vascular SMCs inducing gene expression patterns associated with atherosclerosis.

**Electronic supplementary material:**

The online version of this article (doi:10.1007/s00109-012-0904-1) contains supplementary material, which is available to authorized users.

## Introduction

Although initially identified as targets in the treatment of asthma, recent findings have brought attention to leukotrienes (LTs) as potential mediators of cardiovascular disease [1], such as atherosclerosis [2], abdominal aortic aneurysms [3], and aortic stenosis [4]. Human coronary artery atherosclerotic lesions are a source of cysteinyl LTs (i.e., LTC_4_, D_4_, and E_4_) [5], and urinary levels of LTE_4_ are increased in acute coronary syndromes [6], implicating these mediators in coronary atherosclerosis and plaque instability. The notion of cysteinyl LTs as potential effectors of atherosclerosis has also received support from animal models showing beneficial effects on atherosclerosis burden [7, 8] and intimal hyperplasia [9] by specific antagonists of the leukotriene CysLT_1_ receptor.

The cysteinyl LTs induce their action through G-protein-coupled receptors referred to as CysLT_1_ and CysLT_2_, and the existence of further subclasses of CysLT receptors also has been suggested [10]. In human carotid atherosclerotic lesions, a 3-fold higher CysLT_1_ receptor expression compared with the CysLT_2_ receptor has been reported [11], although the cellular localization of these receptors has not yet been resolved. The signaling through the CysLT_1_ receptor subtypes have been widely studied in the context of bronchoconstriction and asthma [10]. Recently, the CysLT_1_ receptor antagonist montelukast was shown to be associated with a decreased risk of ischemic stroke and a decreased risk of myocardial infarction in males [12]. Although the latter report provides a first indication of beneficial effects of clinically used anti-leukotriene drugs, the exact role of CysLT receptor signaling in atherosclerosis remains to be established.

In addition to being bronchoconstrictors, cysteinyl LTs are also potent vasoconstrictors in the human lung [13]. Their role in the coronary vasculature, however, can be said to be contextually antithetical, as healthy human coronary arteries are unresponsive to cysteinyl LTs, but a contractile response to either LTC_4_ or LTD_4_ is observed in atherosclerotic coronaries [14, 15]. Although no previous study has addressed the mechanism for this differential sensitivity between healthy and atherosclerotic vessels, it is interesting to note that these leukotriene-induced contractions are inhibited by CysLT_1_ receptor antagonists [14]. The latter observation is, however, in contrast to the dominant CysLT_2_ receptor expression in human coronary artery smooth muscle cells (SMCs) [16].

With the notion in mind that a local production of cysteinyl LTs within the atherosclerotic lesion [5] could potentially activate CysLT receptors within the vascular wall, we engaged in this study with the hypothesis that the inflammatory environment of atherosclerosis could lead to an upregulation of CysLT_1_ receptors on vascular SMCs. Here, we have investigated this idea, with the aim to quantify and describe the nature of the increased CysLT_1_ signaling. Further, we have explored the potential downstream effects of this phenomenon on both intracellular signaling and gene expression in vascular SMCs in an effort to link CysLT_1_ receptor signaling to atherogenic properties of SMCs in the context of inflammation and leukotriene stimulation.

## Methods

### Cell culture

Human coronary artery SMC purchased from Clonetics (Cambrex Bio Science, Walkersville, MD, USA) were cultured in SmGM2 kit medium as previously described [17] and harvested for experiments between passages 5 and 8. Cells were seeded in six-well plates (10^5^ cells/well) containing SmGM2 48 h before the respective treatment, which was replaced with Dulbecco’s modified Eagle’s medium supplemented with 2 % fetal calf serum (starvation medium) 24 h before experiments. Subsequently, cells were incubated in the absence or presence of lipopolysaccharide (LPS; from Sigma, 10 μg/mL), interleukin-6 (IL-6; from Peprotech, 20 ng/mL), interferon-γ (IFN-γ; from Peprotech, 20 ng/mL), or tumor necrosis factor-α (TNF-α; from Peprotech, 10 ng/mL). Duration of treatment was experiment-dependent, as described in detail below. Cells were treated with various substances across experiments, including LTC_4_, LTD_4_, and LTE_4_ (from Cayman Chem, Ann Arbor, MI, USA; 1 μM), the CysLT_1_ receptor antagonist MK571 (from Cayman; 1 μM), ethylene glycol tetraacetic acid (EGTA; from Sigma, 5 mM), and 1,2-bis(2-aminophenoxy)ethane-*N*,*N*,*N*′,*N*′-tetraacetic acid tetrakis(acetoxymethyl ester) (BAPTA-AM; from Invitrogen, 10 μM).

### Immunostainings

Atherosclerotic vascular tissue was collected from eight patients undergoing carotid endarterectomy. All experiments were performed in accordance with the ethical standards laid down in the 1964 Declaration of Helsinki and were approved by the local ethics committee (reference number 02/147). All persons gave their informed consent prior to their inclusion in the study. Immunofluorescent stainings were performed on acetone-fixed frozen sections of carotid endarterectomies and on human coronary artery SMCs cultured in LabTek slides after fixation and permeabilization with acetone–methanol. Rabbit anti-human CysLT_1_ receptor (from Cayman Chem) and mouse anti-human α-smooth muscle actin (from DAKO) were used as primary antibodies. Isotype-specific either DyLight 594 or DyLight 488-conjugated secondary antibodies (from Vector) were used, and the nuclei were counterstained with 4′, 6-diamino-2-phenylindol (DAPI; Vector). Images were captured with confocal microscope Leica DMI.

### Calcium signaling experiments

SMCs incubated for 48 h in the absence or presence of LPS (10 μg/mL) were washed and loaded with the fluorescent Ca^2+^ indicator fluo-3. The cells were subsequently stimulated with LTC_4_ (1 μM, 30 min at room temperature) in either Tyrode’s solution (composition, millimolars: NaCl 121, KCl 5.0, NaHCO_3_ 24, CaCl_2_ 0.5, MgCl_2_ 0.4, NaH_2_PO_4_ 0.4, EDTA0.1, and glucose 5.5) gassed with 5 % CO_2_ in O_2_ or Tyrode’s solution containing the CysLT_1_ receptor antagonist MK571 (1 μM). Changes in [Ca^2+^] were recorded using a BioRad MRC 1024 confocal microscopy unit attached to a Nikon Diaphot 200 inverted microscope with a Nikon Plan Apo ×20 or ×60 oil immersion objective (N.A.1.3), as previously described [18].

### RNA extraction, cDNA synthesis, and TaqMan real-time PCR

Total RNA was extracted using RNeasy Mini kit (from Qiagen, Hilden, Germany) with an on-column DNase digestion step. RNA quantity was assessed using a Nanodrop ND-1000 microvolume spectrophotometer (Thermo Fisher Scientific), and RNA quality was assessed by a Bioanalyzer capillary electrophoresis system (Agilent Technologies, Palo Alto, CA, USA). First-strand cDNA was synthesized using Superscript II (Invitrogen, Carlsbad, CA, USA) with random hexamers according to the manufacturer’s instructions. Quantitative TaqMan PCR was performed on a 7900HT Fast Real-Time PCR system (Applied Biosystems) with primer/probe pairs that were obtained using Assay-on-demand™ from Applied Biosystems for human CysLT_1_ (Hs00272624_s1) and Plasminogen Activator Inhibitor 2 (PAI-2/SERPIN B2; Hs01010736_m1). Levels of mRNA were normalized to expression levels of cyclophilin A (Hs99999904_m1), which previously has been determined as an appropriate housekeeping gene in these cells [17]

### Microarray analysis

Microarray analysis experiments were performed on RNA derived from three separate SMC culture experiments, using either Agilent one-color whole human genome (44 K) kit (Agilent Technologies, Redwood City, CA, USA; *n* = 2) or the Affymetrix Human Genome U133 Plus 2.0 array (*n* = 1). Microarrays were analyzed with the Agilent high-resolution microarray scanner. Data were subsequently uploaded to GeneSpring GX10 (Agilent technologies) and analyzed using advanced analysis workflow for the Agilent one-color arrays. The set of data was normalized according to recommendations by GeneSpring for one-color arrays. (http://www.chem.agilent.com/cag/bsp/products/gsgx/manuals/GeneSpring-manual.pdf). Variability between chips was accounted for by applying a shift to the 75th percentile (dividing all measured signals by a 75th percentile value). Per-gene normalization was performed by bringing the baseline to the median of all samples. Probe sets were firstly filtered by confidence of detection, where genes that were not confidently detected in any sample were excluded from further analysis. Further filtering based on expression discarded any genes where less than 100 % of samples in either the relapse or diagnosis condition had expression values below the 20th percentile. The most differentially expressed genes, defined as those with an uncorrected *P* value of <0.05 and demonstrating a fold change in expression of 2.0 or greater, were selected for analysis. The list of 90 genes generated was subsequently compared to data from the Affymetrix arrays, and the genes of interest were verified in terms of direction of regulation. Genes meeting all these criteria are presented in Table 1. The 45 genes listed were submitted to Ingenuity® pathway analysis for prediction of canonical pathways and functional gene networks affected by the significant differential expression of these genes.

**Table 1:** Most significantly differentially expressed genes in response to LTC_4_ (1 μM) in LPS-primed human coronary artery SMCs (sorted by fold change)

Gene ID	Probe ID	Gene name	Mean fold change	Direction	*P* value
CSF3	A_23_P501754	Colony stimulating factor 3	15.88	Up	0.04128
IL24	A_23_P51951	Interleukin 24	15.11	Up	0.04918
SERPINB2	A_23_P153185	Serine proteinase inhibitor 2 (plasminogen activator inhibitor 2)	5.46	Up	0.03882
NEFM	A_24_P264832	Neurofilament medium polypeptide	4.89	Up	0.01537
GJA1	A_23_P93591	Gap junction protein. Alpha 1	4.74	Up	0.00931
IL1A	A_23_P72096	Interleukin 1 alpha	4.15	Up	0.01096
CXCR4	A_23_P102000	Chemokine (C-X-C motif) receptor 4	3.94	Up	0.00170
TFPI2	A_24_P95070	Tissue factor pathway inhibitor 2	3.90	Up	0.02599
CCND1	A_24_P124550	Cyclin D1	3.28	Up	0.04980
CXCL3	A_24_P183150	Chemokine (C-X-C motif) ligand 3	3.27	Up	0.02149
ABCG1	A_23_P166297	ATP-binding cassette. Sub-family G. Member 1	3.03	Up	0.00178
HAS1	A_23_P27400	Hyaluronan synthase 1	2.85	Up	0.03572
MMP3	A_23_P161698	Matrix metallopeptidase 3	2.70	Up	0.01282
PITPNC1	A_24_P772103	Phosphatidylinositol transfer protein. Cytoplasmic 1	2.55	Up	0.03758
KITLG	A_23_P204654	KIT ligand	2.49	Up	0.00730
FUBP3	A_23_P435833	Far upstream element (FUSE) binding protein 3	2.46	Up	0.00619
NPTX1	A_23_P124905	Neuronal pentraxin I	2.45	Up	0.00504
PCSK1	A_23_P213508	Proprotein convertase subtilisin/kexin type 1	2.41	Up	0.04376
ADSS	A_23_P859	Adenylosuccinate synthase	2.40	Up	0.01023
TFPI2	A_23_P393620	Tissue factor pathway inhibitor 2	2.38	Up	0.01401
NPR3	A_23_P327451	Natriuretic peptide receptor C/guanylate cyclase C	2.33	Up	0.01309
SPRED1	A_23_P54460	Sprouty-related. Evh1 domain containing 1	2.30	Up	0.02536
PITPNC1	A_23_P84189	Phosphatidylinositol transfer protein. Cytoplasmic 1	2.29	Up	0.01626
NKAIN1	A_23_P51376	Na+/K+ transporting ATPase interacting 1	2.26	Up	0.02838
SOCS4	A_24_P90637	Suppressor of cytokine signaling 4	2.23	Up	0.00918
TMTC3	A_24_P944222	Transmembrane and tetratricopeptide repeat containing 3	2.22	Up	0.04956
CACNG8	A_32_P61693	Calcium channel. Voltage-dependent. Gamma subunit 8	2.19	Up	0.00480
RRN3	A_23_P206877	RNA polymerase I-specific transcription initiation factor RRN3	2.15	Up	0.00613
APOBEC3F	A_23_P369966	Apolipoprotein B mRNA editing enzyme. Catalytic polypeptide-like 3F	2.12	Up	0.03633
NFE2L3	A_24_P136653	Nuclear factor (erythroid-derived 2)-like 3	2.08	Up	0.00912
MYH11	A_24_P70183	Myosin heavy chain 11 (smooth muscle)	10.44	Down	0.02218
MYH11	A_23_P206920	Myosin heavy chain 11 (smooth muscle)	9.66	Down	0.01751
SCRG1	A_23_P167159	Stimulator of chondrogenesis 1	4.73	Down	0.01807
TPD52L1	A_23_P31143	Tumor protein D52-like 1	4.64	Down	0.01830
CARD9	A_23_P500433	Caspase recruitment domain family. Member 9	3.54	Down	0.00009
CLIC3	A_23_P254654	Chloride intracellular channel 3	3.53	Down	0.00765
KRT7	A_23_P381945	Keratin 7	3.37	Down	0.03569
PCK2	A_23_P128817	Phosphoenolpyruvate carboxykinase 2 (mitochondrial)	2.66	Down	0.02696
CTAGE1	A_24_P124805	Cutaneous T cell lymphoma-associated antigen 1	2.57	Down	0.01412
GLI1	A_23_P105251	GLI family zinc finger 1	2.56	Down	0.01105
SLC1A7	A_23_P325562	Solute carrier family 1. Member 7	2.53	Down	0.00758
TGM1	A_23_P65618	Transglutaminase 1	2.45	Down	0.00203
IL17RD	A_32_P188860	Interleukin 17 receptor D	2.39	Down	0.01570
ZNF467	A_23_P59470	Zinc finger protein 467	2.19	Down	0.00619
LIMS2	A_23_P142796	LIM and senescent cell antigen-like domains 2	2.05	Down	0.01940

Note that some genes may be listed twice due to significant differences detected by independent probes

### ELISA

PAI-2 ELISA was carried out on supernatants from untreated SMCs and SMCs treated LTC_4_ (1 μM) for 24 h using IMUBIND® PAI-2 ELISA kit (from American Diagnostica GmBH, Pfungstadt, Germany) according to manufacturer’s protocol.

### Data analysis

All results are expressed as mean±SE. Statistically significant differences were determined by either a Student’s *t* test (for pair-wise comparisons) or a one-way analysis of variances, followed by Holm–Sidak post hoc test, for multiple comparisons, using Sigma Stat software. A *P* value of less than 0.05 was considered significant.

## Results

### CysLT_1_ receptor expression on vascular SMC

**Fig. 1:**
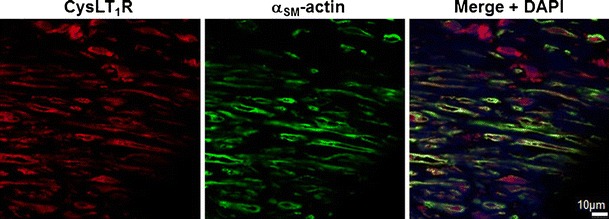
CysLT_1_ receptor expression in human atherosclerotic lesions. Representative immunofluorescent staining of human atherosclerotic plaques from carotid artery showing colocalization of the CysLT_1_ with α-smooth muscle actin-positive vascular smooth muscle cells. Original magnification, ×40

**Fig. 2:**
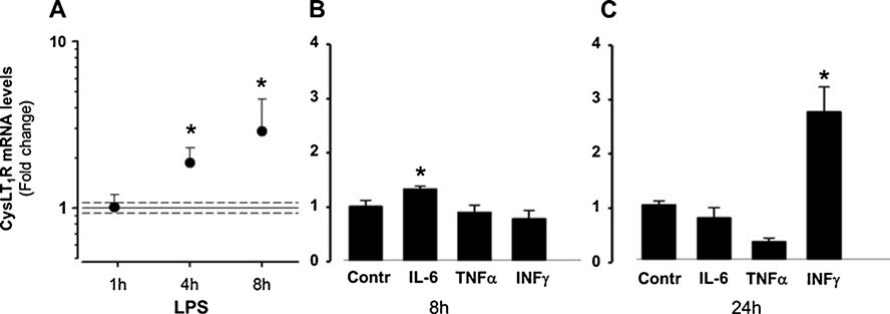
CysLT_1_ receptor expression in human coronary artery smooth muscle cells is upregulated by pro-inflammatory stimuli. Real-time quantitative TaqMan RT-PCR for CysLT receptor mRNA in SMCs incubated in the absence and presence of LPS (10 μg/mL) for 1, 4, and 8 h (**a**) and IL-6 (20 ng/mL), TNF-α (10 ng/mL), or IFN-γ (20 ng/mL) for either 8 h (**b**) or 24 h (**c**). Results are expressed as fold increase compared with untreated cells (*n* = 3–5). *P < 0.05 vs. time-matched control

**Fig. 3:**
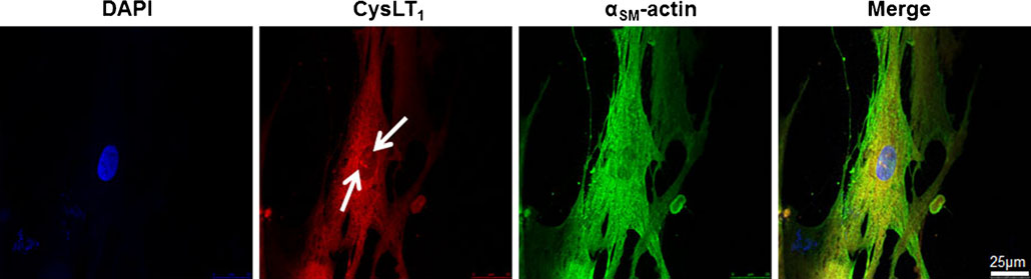
Perinuclear CysLT_1_ receptor expression in human coronary artery smooth muscle cells. Fluorescent labeling of CysLT_1_ receptor protein (DyLight 594 *red* chromogen) and α_SM_-actin (DyLight 488 *green* chromogen) in SMCs. Nuclei were stained with DAPI. *Arrows* indicate nuclear inclusions. Original magnification, ×63

Immunohistochemical staining showed colocalization of the CysLT_1_ receptor protein with markers for SMC (α-smooth muscle actin) in human atherosclerotic lesions (Fig. 1). In human coronary artery SMCs, the transcriptional levels of the CysLT_1_ receptor were time-dependently increased by LPS, IL-6, and IFN-γ (Fig. 2). Fluorescent immunostainings revealed a predominantly perinuclear localization of the CysLT_1_ receptor in human coronary artery SMCs compared with α-smooth muscle actin, which stained positive in the whole cytoplasm (Fig. 3). The CysLT_1_ receptor in some cases demonstrated nuclear inclusions, as indicated by arrows in Fig. 3.

### LTC_4_-induced nuclear calcium signaling in vascular SMCs

**Fig. 4:**
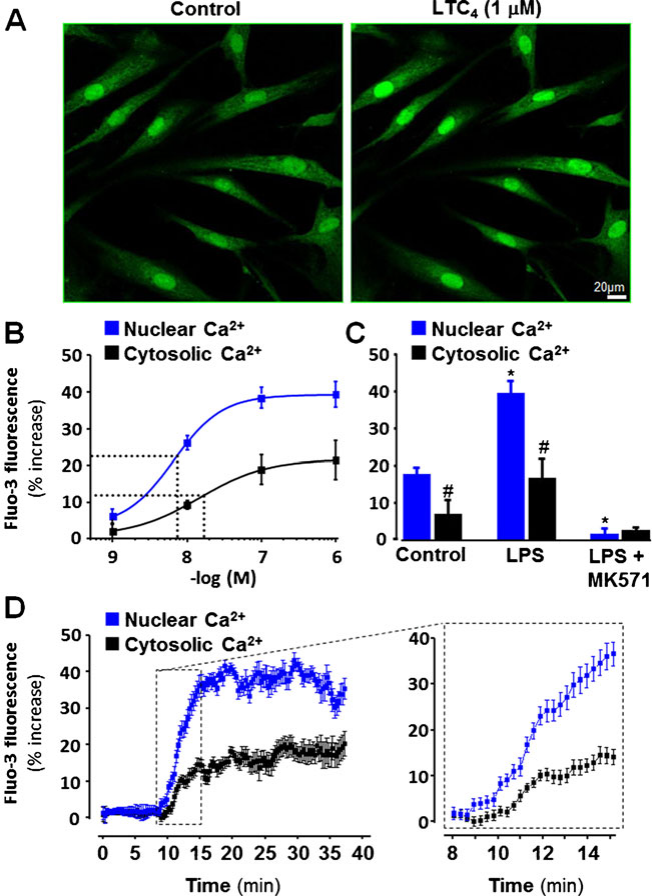
LTC_4_-induced calcium signaling in human coronary artery SMC. **a** Representative micrographs of Ca^2+^ fluorescence in the absence and presence of LTC_4_ (1 μM). **b** Concentration–response curves for Ca^2+^ fluorescence in nuclei (*blue symbols*) and cytosol (*black symbols*) of SMCs incubated for 48 h in the presence of LPS (10 μg/ml). **c** Ca^2+^ fluorescence in nuclei (*blue bars*) and cytosol (*black bars*) of SMCs incubated for 48 h in the absence (control) or presence of LPS (10 μg/ml) prior to stimulation with LTC_4_ (1 μM, 30 min). **d** The time course of the LTC_4_-induced calcium increase shows that the increase in nuclear calcium (*blue symbols*) preceded the increase in cytosolic calcium (*black symbols*). *P < 0.05 vs. controls, ^*#*^ *P* < 0.05 vs Nuclear Ca^2+^

To evaluate whether CysLT_1_ receptors expressed on vascular SMC were functional, calcium changes in human coronary artery SMC were studied using the fluorescent Ca^2+^ indicator fluo-3 (Fig. 4a). LTC_4_ induced a dose-dependent increase in intracellular calcium, which was predominantly located in the nucleus (Fig. 4b). The LTC_4_-induced calcium increase was significantly higher in LPS-treated cells compared with untreated cells (Fig. 4c). In LPS-treated cells, the LTC_4_-induced increase in nuclear calcium was significantly inhibited by the CysLT_1_ receptor antagonist MK571 (Fig. 4c). The time course of the LTC_4_-induced calcium increase in the nuclear and cytosolic compartments is shown in Fig. 4d. The increase in nuclear calcium preceded the increase in cytosolic calcium (Fig. 4d).

### LTC_4_-induced gene expression in vascular SMCs

**Fig. 5:**
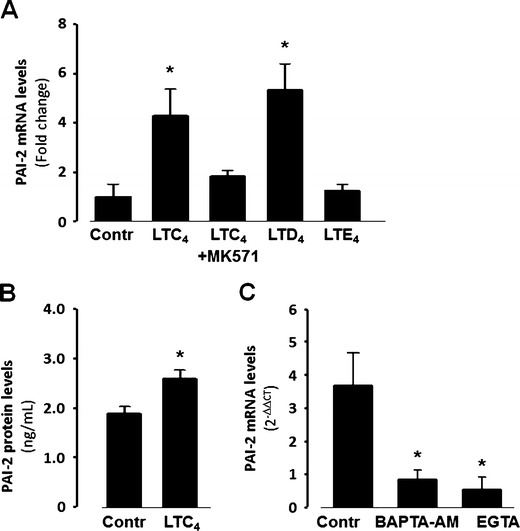
LTC_4_-induced upregulation of PAI-2 in human coronary artery SMC. **a** Real-time quantitative RT-PCR for PAI-2 mRNA in human coronary artery SMC incubated in the absence and presence of LTC_4_, LTD_4_ or LTE_4_ (1 μM) for 24 h. In some experiments, cells were pretreated with the CysLT_1_ receptor antagonist MK571 (1 μM) for 1 h before addition of LTC_4_. *P < 0.05 vs. non-LTC_4_ stimulated Contr (*n* = 3–6). **b** PAI-2 concentrations in supernatants from human coronary artery SMC incubated 24 h in the absence (*Contr*) or presence of LTC_4_ (1 μM). *P < 0.05 vs. Contr (*n* = 7). **c** Increase in PAI-2 mRNA levels induced by LTC_4_ (1 μM) in human coronary artery SMC incubated in the absence (*Contr*) or presence of either BAPTA-AM or EGTA. *P < 0.05 vs. Contr (*n* = 3–6)

The genes most significantly differentially expressed in response to LTC_4_ (1 μM) in LPS-primed human coronary artery SMCs are presented in Table 1. PAI-2 (SERPIN B2), a member of the serine protease inhibitor superfamily, presented as one of the most significantly upregulated genes in the microarray analysis. This finding was confirmed by quantitative PCR (Fig. 5a), and in addition, increased PAI-2 protein levels were detected in the supernatant derived from LTC_4_-stimulated human coronary artery SMCs, compared with unstimulated cells (Fig. 5b). The increased mRNA levels of PAI-2 induced by LTC_4_ (1 μM) were mimicked by LTD_4_ (1 μM) but not LTE_4_ (1 μM) and significantly inhibited by the CysLT_1_ receptor antagonist MK 571 (1 μM; Fig. 5a). In addition, the LTC_4_-induced increase in PAI-2 mRNA was abolished by the removal of intra- and extracellular calcium, through experiments performed in the presence of BAPTA-AM and EGTA (Fig. 5c).

Ingenuity pathway analysis identified several functional gene networks predicted to be significantly affected by LTC_4_ stimulation as based on the 45 genes determined to be differentially expressed through our microarray analysis (Table 1). The highest-scoring network (with a score of 35, equating to a fishers’ exact test score of 1 × 10^−35^) containing 15 genes from Table 1 is suggested to be implicated in cellular movement and hematopoietic system function and development. An outline of the network is shown in Supplementary Fig. 1.

## Discussion

The results of the present study showed an upregulation of predominantly perinuclear CysLT_1_ receptors in vascular SMCs under inflammatory conditions which was associated with increased nuclear calcium signaling and changes in gene expression. Taken together, these findings suggest a role of nuclear CysLT_1_ receptor signaling in vascular SMCs inducing gene expression patterns associated with atherosclerosis.

Previous studies have suggested a dominant expression of the leukotriene CysLT_2_ receptor subtype in human coronary arteries [16]. In the present study, priming of vascular SMCs with LPS upregulated CysLT_1_ receptor mRNA and enhanced LTC_4_-induced effects. Similar findings have been reported in endothelial cells, which under resting conditions exhibit a dominant CysLT_2_ receptor, but in which prolonged exposure to LPS or pro-inflammatory cytokines upregulate CysLT_1_ receptor expression [19]. In the present study, CysLT_1_ receptor expression was also upregulated in SMCs by IL-6 and by prolonged exposure to IFN-γ. Taken together, these observations suggest that a pro-inflammatory environment, such as atherosclerosis, may induce CysLT_1_ receptor expression within the vascular wall. In support of the latter notion, it has been shown that LTC_4_ induces contractions of atherosclerotic but not healthy coronary arteries [14, 15] and that CysLT_1_ receptor signaling, but not CysLT_2_ receptor signaling, is coupled to vasoconstriction in isolated systemic vessels [20]. LTC_4_ has also been associated with SMC proliferation and the shift of SMCs into a synthetic phenotype [21], which is in line with findings that CysLT_1_ receptor antagonism inhibits intimal hyperplasia after vascular injury in mice [9].

The present study is the first demonstration of a perinuclear localization of functional CysLT_1_ receptors in vascular SMCs and a leukotriene-induced nuclear calcium signaling in these cells. These findings are nevertheless consistent with other G-protein-coupled receptors in vascular SMCs. For example, the ET_A_ endothelin receptor and the AT_1_ angiotensin II receptor exhibit a perinuclear localization in vascular SMCs coupled to nuclear calcium signaling [22–24]. In addition, we have recently demonstrated a perinuclear localization of the CysLT_1_ receptor in valvular interstitial cells derived from human aortic valves [4], corroborating prior observations of CysLT_1_ receptor expression at the outer nuclear membrane in intestinal epithelial cells [25]. In both of these cell types, leukotriene stimulation induces an increase in nuclear calcium [4, 25]. The present study extends those findings by demonstrating an enhanced nuclear calcium increase in response to LTC_4_ after priming of cells with LPS and that the increase in nuclear calcium preceded the increase in cytosolic calcium. Whereas the present study cannot definitely conclude whether the subcellular CysLT_1_ receptor localization represents an internalization process, previous studies support a translocation of CysLT_1_ receptors between different cellular compartments, including nuclear inclusions [26].

Nuclear calcium is a key regulator of gene expression [27], and in line with this notion, LTC_4_ induced significant changes in expression of several genes in the present study. Of particular interest was the appearance of PAI-2 as one of the most significantly upregulated genes in the microarray analysis. This was confirmed with qPCR analysis, and ELISA measures in addition showed that PAI-2 protein secretion from human coronary artery SMCs was increased by LTC_4_ stimulation. PAI-1 and PAI-2 are members of the serine proteinase inhibitor family and act as important inhibitors of fibrinolysis by interfering with the plasminogen system. PAI-1 is induced by atherogenic stimuli in vascular SMCs and may participate in cell growth and matrix degradation associated with atherosclerosis [28]. In addition, using cDNA representational difference analysis, PAI-2 has previously been identified as one of the most differentially expressed genes in atherosclerotic lesions compared with normal vessels, with elevated PAI-2 expression preferentially observed in unstable carotid plaques [29]. In addition, another study using serial analysis of gene expression in human vascular SMCs also identified PAI-2 as one of the most upregulated genes in response to conditioned media derived from macrophages activated by oxidized low-density lipoprotein [30]. Finally, immunohistochemical analysis of human atherosclerotic lesions has also confirmed that vascular SMCs stain positive for PAI-2 [29, 30]. In addition to acting as a plasminogen activator inhibitor, PAI-2 may serve as a regulator of Th1 immune responses through the modulation of cytokine-induced responses [31]. Furthermore, PAI-2 may be associated with the process of wound healing post-plaque rupture [29].

The LTC_4_-induced increase in PAI-2 mRNA was abolished when experiments were performed in the presence of calcium chelators, suggesting a calcium dependent upregulation of PAI-2. The latter notion is supported by previous studies showing that angiotensin II is a potent inducer of PAI-2 in vascular SMCs through the AT_1_ receptor [32], which is in line with a perinuclear AT_1_ receptor localization and an EGTA-sensitive nuclear calcium signaling induced by angiotensin II [33].

Ingenuity pathway analysis revealed a significant number of LTC_4_-upregulated genes to be implicated in a functional gene network linked to hematopoietic system function and cellular movement. Of note in this network was the involvement of cAMP response element binding protein (CREB), a transcription factor and member of the leucine zipper family of DNA binding proteins, which is known to be activated by nuclear calcium [27]. Furthermore, the PAI-2 gene promoter region contains a binding site for CREB (−1,319 bp), and this transcription factor has been shown to be associated with the induction of PAI-2 expression [34]. Taken together, these findings suggest that LTC_4_-induced changes in gene expression may be induced through an increase in nuclear calcium leading to CREB activation. However, the pathway analysis also revealed other pathways that may be involved in LTC_4_-induced gene expression, such as the NF-κB signaling pathway which has been previously shown to be activated after CysLT_1_ receptor ligation in leukocytes [10].

In summary, we have shown that pro-inflammatory stimulation of vascular SMCs enhances perinuclear CysLT_1_ receptor expression coupled to nuclear calcium signaling and results in changes in gene expression, such as upregulation of PAI-2. Since cysteinyl-LT production is increased in atherosclerosis [5] and acute coronary syndromes [6], an altered vascular sensitivity to leukotriene-induced SMC gene expression secretion may further enhance the inflammatory response. As such, targeting CysLT_1_ receptors could potentially be of therapeutic interest in atherosclerosis.

## Electronic supplementary material

Below is the link to the electronic supplementary material.

ESM 1 (PDF 194 kb)
