# Supplementary material for: Cysteinyl leukotriene signaling through perinuclear CysLT1 receptors on vascular smooth muscle cells transduces nuclear calcium signaling and alterations of gene expression
Source: J Mol Med (Berl). 2012 Apr 20;90(10):1223–31. doi: 10.1007/s00109-012-0904-1 (PMC3438394; doi:10.1007/s00109-012-0904-1)
Supplement: Supplementary file 1 — (PDF 194 kb) [file 109_2012_904_MOESM1_ESM.pdf]

Eaton et al.

Cysteinyl leukotriene signaling through perinuclear CysLT$_1$ receptors on vascular smooth muscle cells transduces nuclear calcium signaling and alterations of gene expression

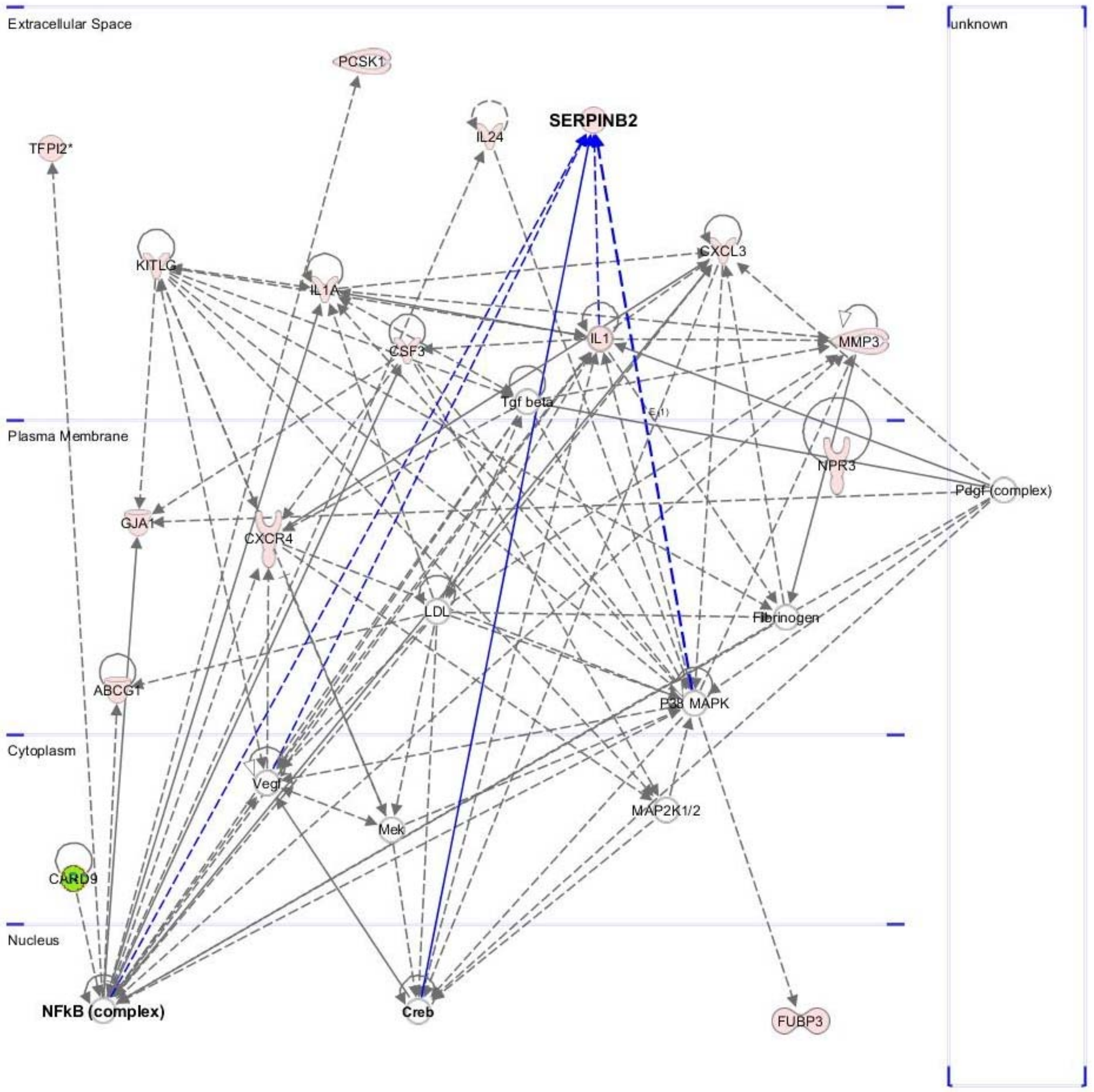

Supplementary Fig 1. ***Functional gene network associated with upregulation by LTC$_4$ stimulation (derived from Ingenuity Pathway analysis).*** Network shown, suggested to be implicated in cellular movement and hematopoietic system function, was found to be markedly upregulated (Fishers' exact test score $1x10^{-35}$) by LTC$_4$ stimulation based on analysis of significantly differentially expressed genes (Table 1). Red shading indicates significantly upregulated genes. Intensity of the shading is proportional to the fold change. Interactions involving PAI-2 are highlighted in blue.
